# Radiation exposure to the urology surgeon during retrograde intrarenal surgery

**DOI:** 10.1371/journal.pone.0247833

**Published:** 2021-03-15

**Authors:** Il woo park, Su Jin Kim, Dongseong Shin, Sung Ryul Shim, Hyun Kyung Chang, Chang Hee Kim

**Affiliations:** 1 Department of Urology, Gil Medical Center, Gachon University College of Medicine, Incheon, Republic of Korea; 2 Department of Urology, Yonsei University Wonju College of Medicine, Wonju, Republic of Korea; 3 Department of Pharmacology, Gachon University College of Medicine, Incheon, Korea; 4 Clinical Trials Center, Gachon University Gil Medical Center, Incheon, Korea; 5 Department of Preventive Medicine, Korea University College of Medicine, Seoul, Republic of Korea; 6 Department of Urology, Catholic Kwandong University, International St. Mary’s Hospital, Incheon, Republic of Korea; IPATIMUP/i3S, PORTUGAL

## Abstract

Retrograde intrarenal surgery is a common procedure that carries a risk of radiation exposure for urologists. This study aimed to measure the amount of radiation that urologists are exposed to during surgery, and to estimate how many procedures can be safely performed by one urologist per year. Variables that affect radiation exposure were also identified. Radiation exposure doses were measured for the eye, neck, chest, arms, and hands of a urologist who performed 226 retrograde intrarenal surgeries. To determine how many procedures could be safely performed per year, the Annual Permissible Occupational Exposure Radiation Dose Guidelines of the National Council on Radiation Protection and Measurements were consulted. Correlations between radiation exposure dose and the patient’s age, sex, body mass index, stone number/burden/laterality/location/Hounsfield unit, and their renal calculi were calculated. The mean surgery and fluoroscopy durations were 83.2 and 5.13 min; the mean tube voltage and current were 68.88 kV and 2.48 mA, respectively. Cumulative radiation doses for the eye, neck, chest, right upper arm, left hand, and right hand were 65.53, 69.95, 131.79, 124.43, 165.66, and 126.64 mSv, respectively. Radiation reduction rates for lead collars and aprons were 97% and 98%, respectively. If the urologists wear only radiation shields and lead apron but do not wear safety glasses during RIRS, the recommended by the ICRP publication 103 is taken into consideration, our results showed that 517 RIRS can be performed per year safely. However, if no protective measures are taken, this number decreases to only 85 RIRS per year. At all measurement sites, significant correlations were observed between the radiation exposure dose and stone numbers and Hounsfield unit values. In conclusion, it is imperative that urologists wear protective gear. Greater effort should be made to reduce radiation exposure when renal calculi have a large number of stones or large Hounsfield unit values.

## Introduction

If the size of a renal calculus is smaller than 20 mm, extracorporeal shockwave lithotripsy (ESWL) is usually considered as the primary means of treatment [1]. However, if the renal calculi are large, located in the lower kidney, or numerous, they do not respond well to treatment and adverse effects can occur if ESWL is performed repeatedly. Therefore, surgical procedures such as retrograde intrarenal surgery (RIRS) or percutaneous nephrolithotomy (PCNL) are considered in these cases [2, 3]. Although PCNL is usually the first line of treatment for renal calculi that are larger than 20 mm [1], some patients may experience severe complications such as hemorrhaging, urinary leakage, intestinal injury, pneumothorax, or hemothorax [4]. Thus, along with the recent development of a flexible endoscope, RIRS is more commonly being considered as the first line of surgical treatment for renal calculi because it does not require transcutaneous invasion and has similar success rates to that of PCNL. When RIRS is performed to remove renal calculi, a fluoroscopic guide is routinely used, and as a result, the performing urologist is faced with the risk of radiation exposure. With an increase in the use of radiological equipment, the risk of radiation exposure associated with these types of equipment is being realized. Many studies have demonstrated a dose-dependent effect between the radiation dose and its accompanying adverse effects [4]. Thus, in many medical fields, efforts are being made to minimize radiation exposure for both patients and radiation workers who are exposed to radiation [5]. However, few studies have examined the risk of radiation exposure to urologists who perform RIRS.

This study aimed to measure the yearly radiation exposure of urologists who perform RIRS and consider the yearly threshold of radiation exposure for medical workers to determine the number of RIRS procedures that is deemed safe for urologists to perform. Furthermore, guidance for reducing the radiation dose during RIRS for urologists was supported by identifying variables that can influence the amount of radiation exposure.

## Materials and methods

### Study design and radiation dose measurement

This study was conducted from October 2018 to September 2019 at the Gil Medical Center with 226 patients who were diagnosed with renal calculi and who received RIRS from a single urologist. Prior to the surgery, patients underwent the following: collection of medical history, physical examination, routine blood and urine tests, plain radiography of the kidney-ureter-bladder, and non-contrast enhanced computed tomography with three-dimensional reconstruction. Based on the radiological findings, stone factors including the stone number, burden, laterality, location, and Hounsfield unit (HU; stone density) were examined. Stone burden was calculated using the renal calculi length and width to find the stone’s surface area (length*width*3.14/4) [6]. The urologist wore a lead apron (0.35-mm lead-equivalent thickness; Bar-ray Inc., USA), knee-length and front protection, and a thyroid shield (0.35-mm lead-equivalent thickness; Bar-ray Inc., USA) and did not wear protective glasses or gloves. While performing the RIRS, an optically stimulated luminescence albedo neutron dosimeter (Landauer Inc., USA) was placed beside the urologist’s right eye; two dosimeters were placed on the urologist’s chest inside the lead apron and thyroid shield, and two dosimeters were placed outside the lead apron and thyroid shield. The effective dose, i.e., the quantity that is related to the stochastic radiation risk, is generally considered to be the amount of radiation that the radiation worker is exposed to [7]. However, it is difficult to realistically measure the effective dose, as it is a weighted sum of doses for several organs. Hence, several algorithms have been proposed to obtain estimates of the effective dose [8–12]. In this study, we used the following formula proposed by Faulkner *et al* to calculate the surgeon’s effective dose: (0.5 × dose for the chest below the lead apron) + (0.025 × dose for the chest above the lead apron) [12]. To measure radiation exposure on the extremities of the urologist, a dosimeter was placed on the right upper arm and on both hands. After study completion, all of the dosimeters were sent to a radiation measuring company (Hanil Nuclear Co., Ltd, Anyang, Korea) to determine the total amount of radiation that the urologist was exposed to.

### RIRS technique with fluoroscopy

Thereafter, the patient was placed in a lithotomy position to insert the ureteroscope and checked for any abnormalities in the bladder and in the orifice of the ureter. The guide wire was inserted using a semi-rigid ureteroscope (9.5 Fr; Olympus, Japan), and the ureteroscope was inserted into the ureter. The ureteroscope was directed into the upper ureter to check for any stenosis, tumor, or any type of lesions. Afterwards, the ureteroscope was relocated to the bladder to check that the ureteral access sheath (UAS, Ureteral Access Sheath, 12/14 Fr, 46–48 cm; Boston Scientific Co., USA) passed through the ureterovesical junction, and following the guide wire, the UAS was placed in the ureteropelvic juncture. Then, the guide wire and the inner obturator were removed, and the flexible ureteroscope (8.5 Fr; Uretero-Reno Flexible videoscope-V2; Olympus, Japan) was inserted into the UAS. When the stone was visible, it was pulverized using a Holimum:YAG laser (Omnipulse-Max™, Trimedyne, USA) and removed using stone forceps or a stone basket. A 365 μm end firing fiber was used for the laser with a 1.0 J/pulse energy setting and a 10 Hz frequency. The ureteral stent was maintained for one week after surgery in all cases to prevent ureteral stricture formation and to reduce the incidence of postoperative renal colic secondary to ureteral edema.

In all surgeries, a fluoroscope (OEC fluorostar 7900, GE, USA) was used, and the X-ray source was located under the patient. The fluoroscope was located to the left of the urologist, as was the monitor of the fluoroscope. The tube voltage (in kV), tube current (in mA), fluoroscopy screening time, and operation time were recorded. The fluoroscope used in this experiment had an automatic brightness control mode, and thus, the optimal tube voltage and current were automatically set. The ranges for tube voltage and current were 60–80 kV and 1.70–2.94 mA, respectively. The urologist performed the procedure sitting down, and all of the dosimeters were set at specific distances from the X-ray source of the fluoroscope (eye [110 cm], chest [95 cm], and neck [100 cm]). Additionally, the urologist made an effort to minimize movement to maintain a set distance from the X-ray source of the fluoroscope with the dosimeters located on the right upper arm and on both hands.

### Annually allowed RIRS cases, variables affecting radiation exposure, and statistics

The surgeon’s effective dose and the levels of radiation measured from the eye and hand were converted into the radiation dose per RIRS case. This was compared with the recommended safe threshold for radiation exposure from the Occupational Exposure Guidelines of the National Council on Radiation Protection and Measurements (NCRP) and the International Commission on Radiological Protection (ICRP) to determine how many RIRS cases can be safely performed in a year [13, 14].

The entrance surface dose (ESD), which is used as the radiation exposure dose of the radiography equipment, utilizes the dosimeters attached on the skin to measure radiation exposure. Jensen et al. conducted a study to compere the lens dose of radiologists in a vascular interventional laboratory using ESD; in this study, we also used the ESD as a means of calculating the dose for the urologists [15]. Mathematical model calculations based on X-ray machine output using the formula given below (1), the Chuan and Tsai formula, can also be used to measure radiation exposure [16–18].

$$ESD=c{\left(\frac{kvp}{FSD}\right)}^{2}\left(\frac{mAs}{mm.AI}\right)$$ (1)

In this formula, kv_p_ represents the X-ray peak tube voltage, and mAs is the exposure value that represents the tube’s current multiplied by the exposure time. Focus to skin distance (FSD) represents the distance between the X-ray tube and the urologist in millimeters. AI is the aluminum filtration, and C is the machine dependent constant [19]. In this study, the peak tube voltage, mean tube current, and the fluoroscopy screening time were substituted into the Chuan and Tsai formula to find the value for each RIRS case. The calculated value was converted into a ratio for each RIRS case and was then applied to the cumulative radiation dose measured by body part of the urologist to determine the radiation dose each part of the urologist’s body was exposed to for each case.

To identify the variables that affect the radiation exposure to the urologist, a multiple linear regression analysis was conducted. The radiation exposure dose from each part of the urologist’s body and the surgeon’s effective dose was the dependent variable in the regression model. The patient’s age, sex, body mass index (BMI), stone number, stone burden, stone laterality, stone location, and HU were the independent variables. The variation inflation factor between the independent variables ranged between 1.055 and 1.443; as a result, multicollinearity was not significant. Statistical analyses were performed using R version 3.5.1 (The R Foundation for Statistical Computing). All statistics are presented as mean ± standard deviations. Two-tailed p-values <0.05 were considered statistically significant.

This study was approved by the Institutional Review Board (No. GAIRB 2020–391) of Gil Medical Center and was conducted in accordance with the principles expressed in the Declaration of Helsinki.

## Results

The average age of the included patients (126 males, 101 females) was 55.74 ± 12.82 years. The mean BMI was 25.68 ± 4.15 kg/m^2^; 99 patients had hypertension (43.8%) and 92 patients had diabetes (40.7%) as an underlying disease (Table 1). The radiation exposure dose for the urologist is listed in Table 2. The cumulative radiation doses were 65.53 mSv for the eye, 69.95 mSv for the neck, 131.79 mSv for the chest, 124.43 mSv for the right arm, 165.66 for the right hand, and 126.64 mSv for the left hand. The cumulative radiation doses inside the lead apron and the thyroid shield were 2.21 mSv and 2.20 mSv, respectively. The cumulative effective dose for the surgeon was 4.39 mSv. The radiation dose per RIRS case was 0.29 mSv for the eye, 0.01 mSv and 0.31 mSv for the inside and outside of the thyroid shield, 0.01 and 0.58 mSv for the inside and outside of the lead apron, 0.02 mSv for the surgeon’s effective dose, 0.55 mSv for the right arm, 0.73 mSv for the right hand, and 0.56 for the left hand, respectively. On comparing the inside and outside of the radiation exposure level of the lead apron and the thyroid shield, radiation reduction percentages of 98% and 97% were found, respectively.

**Table 1:** Demographics of patients, clinical data, and characteristics of stones.

Variables	Value^a^	
**Demographic parameters**		
Age (years)	55.74 ± 12.82	
Sex (Male)	125 (55.31%)	
BMI (kg/m^2^)	25.68 ± 4.15	
Serum creatinine (mg/dL)	0.88 ± 0.35	
Comorbidity		
Hypertension	99 (43.81%)	
Diabetes mellitus	92 (40.71%)	
**Characteristics of stones**		
Stone numbers	1.87 ± 1.08	
Stone burden (mm^2^)	124.21 ± 167.7	
Stone laterality (Right)	92 (40.71%)	
Hounsfield unit	970.09 ± 358.78	
Stone location		
Renal Pelvis	96 (42.48%)	
Upper calyx	21 (9.29%)	
Middle calyx	24 (10.62%)	
Lower calyx	85 (37.61%)	
**RIRS treatment**		
Operation time (min)	83.2 ± 37.24	
Fluoroscopy screening time (min)	5.13 ± 4.59	
Tube voltage (kV)	68.88 ± 3.88	
Tube current (mA)	2.48 ± 0.2	
Hospitalization (days)	3.11 ± 1.78	
Double J ureteral stent indwelling duration		6.97 ± 2.0

a. Data are presented as mean ± standard deviation or median (range) for continuous variables, and as N (%) for categorical variables. Abbreviations: RIRS, retrograde intrarenal surgery; BMI, body mass index.

**Table 2:** Cumulative radiation exposure parameters.

Dosimeter position	Cumulative radiation dose (mSv)	Radiation dose per case (mSv)
Eye	65.53	0.29
Neck	69.95	0.31
Neck, protected	2.21	0.01
Chest	131.79	0.58
Chest, protected	2.21	0.01
Surgeon’s effective dose	4.40	0.02
Upper arm, right	124.43	0.55
Ring finger, right	165.66	0.73
Ring finger, left	126.64	0.56

Dose effective = (0.5 × Dose below the lead apron) + (0.025 × Dose above the lead apron)

According to the recommended occupational exposure limit from the NCRP and ICRP [13, 14], urologists without a whole-body lead apron can only perform 85 RIRS. However, if equipped with a whole-body lead apron, urologists can perform 517 RIRS per year without a safety glass or protect (Table 2).

The average stone number was 1.87 ± 1.08, and the mean stone burden was 124.21 ± 167.7 mm^2^. A left renal stone was found in 134 patients (59.29%), a right renal stone was found in 92 patients (40.71%), a renal pelvis stone was found in 96 patients (42.48%), an upper calyx stone was found in 21 patients (9.29%), a middle calyx stone was found in 24 patients (10.62%), and a lower calyx stone was found in 85 patients (37.61%). The mean HU of the renal calculi was 970.09 ± 358.78. The average operation time was 83.2 ± 37.24 min, while the average fluoroscopy screening time was 5.13 ± 4.59 min. The average tube voltage and current were 68.88 ± 3.88 kV and 3.11 ± 1.78 mA, respectively. The average length of the hospital stay was 3.11 ± 1.78 days, and the ureter stents were generally removed after 6.97 ± 2.0 days.

According to the results of the multiple linear regression analysis, which was conducted to determine the effects of radiation exposure on the urologist during RIRS, the stone number and HU were statistically significant variables for all body parts of the urologist. In particular, as the stone number and HU increased by 1, the radiation doses in the eye increased significantly by 0.053 mSv and 0.055 mSv, and the radiation doses for the right hand increased significantly by 0.133 mSv and 0.139 mSv, respectively (Table 3).

**Table 3:** Variables that affect radiation exposure assessed using multivariate logistic regression analysis.

Dosimeter position	Variables	Coefficient	95% CI Low	95% CI High	p-value
Eye	Age	0.002	<0.001	0.004	0.092
	BMI	-0.053	-0.112	0.006	0.078
	Stone numbers	0.053	0.027	0.078	<0.001
	Hounsfield unit	0.055	0.002	0.109	0.044
Chest	Age	0.004	-0.001	0.008	0.090
	BMI	-0.106	-0.225	0.013	0.082
	Stone numbers	0.105	0.054	0.157	<0.001
	Hounsfield unit	0.111	0.003	0.219	0.046
Surgeon’s effective dose	Age	<0.001	<0.001	<0.001	0.090
	BMI	-0.004	-0.008	<0.001	0.083
	Stone numbers	0.004	0.002	0.005	0.000
	Hounsfield unit	0.004	<0.001	0.007	0.046
Ring finger, right	Age	0.005	-0.001	0.010	0.092
	BMI	-0.132	-0.282	0.017	0.085
	Stone numbers	0.133	-0.117	0.382	<0.001
	Hounsfield unit	0.139	0.003	0.274	0.046

Abbreviations: CI, confidence interval; BMI, body mass index

## Discussion

This study demonstrated that a large number of stones as well as higher HU values exposed the urologist to higher radiation doses during RIRS. Furthermore, considering the yearly recommended exposure limit to radiation, the percentage of RIRS that could be performed safely without radiation shields was only 3.3% compared to those performed with shields. The right hand of the urologist was exposed to the highest radiation dose compared to other body parts (1.3 to 75 times higher).

Several studies have already shown that occupational radiation exposure and different types of cancer are closely related [20–23]. Nuclear power industry workers are at high risk of developing leukemia [24], and medical diagnostic radiographers have a significantly elevated risk of cancer, such as leukemia, skin cancer, breast cancer, lung cancer, liver cancer, bladder cancer, and even esophageal cancer [23]. However, as the use of radioactive technology allows for more accurate diagnosis and more appropriate forms of treatment, experts in the medical field are currently widely using radioactive technology, even with these associated risks. In the 2009 NCRP report 160, in the 1980s, the ionizing radiation exposure comprised mostly natural background radiation (83%), and medical radiation only represented 15% of the total radiation exposure. However, in 2006, the level of radiation emitted from the medical field greatly increased to 50% [25]. This increase was generally explained by the increased use of computed tomography scans, although fluoroscopy and procedures that require its use also played a role in this increase. Since with fluoroscopy the same body part is exposed for a long period of time, it may be more important to find ways to reduce radiation exposure, especially for the skin. ICRP report 85 reports on the dosage of radiation received in fluoroscopy use. It states that the radiation dose that medical staff are exposed to consists mainly of direct exposure to the hand, scattered rays from the patient and the operating table, as well as a small dosage from X-ray leakage [26]. Several studies have shown that interventional procedures that use fluoroscopy can lead to radiation-caused development of cancer [27], especially brain tumors, blood cancer, and lymphoma [28, 29]. Hence, the Occupational Exposure Guidelines of the NCRP and ICRP recommend a yearly occupational exposure limit of 50 mSv for the body, 150 mSv for the eye, and 500 mSv for the skin, hands, and feet [13, 14].

Due to an increase in the use of RIRS for the treatment of renal calculi, the use of fluoroscopic guide associated with RIRS will also increase, thereby increasing the dose of radiation that urologists will be exposed to. Thus, measures need to be taken to reduce the dose of radiation that the urologists are exposed to. This experiment took into consideration the occupational exposure limit to determine the number of RIRS procedures that can be safely performed in a year. If the urologists wear only radiation shields and lead apron but do not wear safety glasses during RIRS, the recommended by the ICRP publication 103 is taken into consideration, our results showed that 517 RIRS can be performed per year safely. However, if no protective measures are taken, this number decreases to only 85 RIRS per year. The radiation dose measured from the right hand, which has no protection from radiation, was 1.3 times higher than that measured from the right upper arm, 38 times higher than the surgeon’s effective dose, and three times higher than that measured from the eye.

Variables that are known to affect the stone-free rate (SFR) in RIRS are the stone size, stone number, HU, and stone location [30, 31]. Among these variables, the stone number has a particularly large effect [32], and patients with a solitary calyceal calculus have a longer RIRS operation time and decreased SFR compared to patients with multiple calyceal calculi [33, 34]. Additionally, the HU of the stone has an effect on the fragmentation efficiency and operation time in RIRS, and as HU increases [35], the operation time [36] as well as the total laser energy and total laser time increases, which thereby decreases the fragmentation efficiency and the SFR [37]. If there are a large number of renal stones or if the HU value is high, the operation time increases, and the fragmentation efficiency decreases while performing RIRS because of the increased washing pressure that leads to increased movement of broken stones, possibly resulting in bleeding in the renal mucosa, which leads to an obstruction of the visual field, causing fatigue for the urologist. If the urologist experiences more fatigue and has an obstructed visual field, they would rely more on the fluoroscopic guide, leading to an increase in operation time, and an increase in fluoroscopy screening time, which results in increased exposure to radiation.

This study shows that, if there are large number of stones or if the HU value is high, the urologist performing the RIRS procedure is at a greater risk of radiation exposure when the automatic radiation exposure setting is used on the fluoroscopy device (Table 3).

As there is an increased use of RIRS for the treatment of renal stones, urologists will also inevitably be exposed to a higher dose of radiation. The results of this study demonstrate that increased exposure to radiation during RIRS occurs if there are a large number of stones or if the HU value is high. Therefore, to protect the urologist from radiation exposure during RIRS, we recommend the following based on the three principles of time-distance-shielding proposed by the ICRP.

To minimize fluoroscopy screening time for RIRS, it is recommended to use an intermittent fluoroscopy technique and pulsed fluoroscopy, which keep the frame rate to a minimum as long as it does not affect surgery. If the fluoroscopy unit is equipped with the feature of leaving the last image on the screen after the beam is turned off, it is recommended to also use the last image hold feature. Additionally, in relation to the results of this study, if patients have high HU stones or a large number of stones that require surgery, imaging tests such as computed tomography scans and kidney-ureter-bladder are recommended prior to surgery to clearly understand the anatomical structure of the patient and to prepare for the surgery beforehand to allow for a quicker and more effective surgery. Further, dosimeters should be worn by all medical staff aiding the RIRS procedure, and staff members who are already near the recommended exposure limit should be excluded from the surgery.

During the RIRS procedure, the urologist should try to maintain maximal distance from the X-ray source, and the distance between the patient and the image receptor should be as close as possible. As the distance between the X-ray source decreases by half, the incident dose increases four times; further, when the distance between the image receptor decreases by half, the incident dose also decreases by half. Additionally, placing the X-ray source in an undercouch system can help reduce the radiation dose, and it is recommended that the source does not leave the undercouch system. As the results in this study showed that the unprotected hand was exposed to the most radiation compared to other parts of the body (up to 1.3 to 75 times more), it is recommended that this hand stays out of the way of the primary beam because of skin damage and increased radiation exposure associated with the primary beam due to automatic radiation exposure control.

When performing surgery with radiologic equipment, the upper extremities of the physician are exposed to radiation; however, the physician’s lower extremities (e.g., groin, knee, back) are also exposed to scatter radiation [38, 39]. Therefore, during RIRS, it is recommended that urologists wear a lead apron that covers them all the way down to their knees in addition to wearing a thyroid shield. If possible, a wrap-around shield is also recommended. According to a study published in 2005, if proper protective equipment are worn during urologic surgeries, the level of radiation exposure can decrease by up to 2% of the yearly radiation limit [40]. In this study, it was also confirmed that the radiation shielding rate of a lead apron and thyroid shield was over 97%. Although there was a high radiation exposure dose on the hands, if protective gloves were worn, there was a 76.6% reduction; thus, wearing gloves is recommended [41]. Additionally, safety glasses can decrease radiation exposure to the eyes by 70–92%, indicating that the use of safety glasses is also very important [42]. Further, it is recommended to use other additional lead shielding equipment: table skirts, rollaway, and ceiling suspended units.

This study has some limitations. First, all medical staff who participate in RIRS surgery are exposed to radiation, however, this study only focused on the urologist’s exposure to radiation. Furthermore, direct exposure to radiations and diffused (reflected) radiation amounts by the patient, wall, and device are considerable, but we could not determine the radiation dose for these, to which the urologists are exposed. Additionally, more studies on various urology surgeries and procedures that also use radiologic equipment other than RIRS are required. We are currently devising additional studies to identify preventive measures to minimize radiation exposure for all medical staff who perform various urological procedures.

## Conclusion

Considering radiation exposure risk, the use of protective gear is necessary to ensure safety and efficacy of RIRS. We believe that efforts to reduce radiation dose before and during surgery are required when renal calculi have large number of stones or larger HU values.

## Supporting information

S1 File Patient’s and clinical data, and radiation exposure parameters. (PDF)
