# Supplementary material for: Radiation exposure to the urology surgeon during retrograde intrarenal surgery
Source: PLoS One. 2021 Mar 15;16(3):e0247833. doi: 10.1371/journal.pone.0247833 (PMC7959394; doi:10.1371/journal.pone.0247833)
Supplement: S1 File — (PDF) [file pone.0247833.s001.pdf]

Number	Age	Sex (Male)	BMI	Comorbidity	Serum creatinine	Stone numbers	Stone burden	Stone laterality	Hounsfield unit	Stone location	Operation time	Fluoroscopy time	Tube voltage	Tube current	Hospitalization	Radiation exposure	Eye	Neck	Neck, protected	Chest	Chest, protected	Upper arm, Right	Ring finger, Right	Ring finger, Left	Surgeon's effective
1	76	0	28.98	3	0.68	2	37.65	1	1041	2	150	9	73	2.72	2.00		0.63	0.67	0.02	1.27	0.02	1.2	1.6	1.22	0.042372241
2	64	1	26.03	1	1.37	3	96.05	0	1296	2	80	6	76	2.72	2.00		0.46	0.49	0.02	0.92	0.02	0.87	1.15	0.88	0.030617635
3	47	0	26.68	0	0.76	4	78.43	1	827	4	112	8	69	2.4	1.00		0.44	0.47	0.01	0.89	0.01	0.84	1.12	0.85	0.029690924
4	50	1	30.6	0	0.75	1	96.78	1	955	1	68.58	6	67	2.4	2.00		0.31	0.33	0.01	0.63	0.01	0.59	0.79	0.6	0.020995992
5	62	0	19.15	1	0.67	2	89.91	0	1212	4	110	8	69	2.4	4.00		0.44	0.47	0.01	0.89	0.01	0.84	1.12	0.85	0.029690924
6	50	1	24.86	1	0.9	4	39.7	0	508	2	86	6	69	2.94	1.00		0.41	0.43	0.01	0.82	0.01	0.77	1.03	0.79	0.027278536
7	44	1	27.86	1	1.17	1	123.02	0	1142	4	68	7	67	2.4	2.00		0.36	0.39	0.01	0.73	0.01	0.69	0.92	0.71	0.024495324
8	56	0	37.78	1	0.69	4	35.99	1	1089	4	76	6	68	2.64	2.00		0.35	0.38	0.01	0.71	0.01	0.67	0.9	0.68	0.023790157
9	69	1	24.86	2	1.68	4	126.15	1	1362	4	120	8	70	2.4	2.00		0.46	0.49	0.02	0.92	0.02	0.86	1.15	0.88	0.030557766
10	35	0	21.1	1	0.22	1	51	1	1024	1	57	3	77	2.7	2.00		0.23	0.25	0.01	0.47	0.01	0.44	0.59	0.45	0.015598785
11	38	1	29.15	0	0.92	1	97.43	1	873	2	69	6	67	2.4	3.00		0.31	0.33	0.01	0.63	0.01	0.59	0.79	0.6	0.020995992
12	57	2	23.23	0	0.64	1	77.95	1	1023	1	83	5	76	2.72	2.00		0.38	0.41	0.01	0.76	0.01	0.72	0.96	0.73	0.025514696
13	73	1	24.1	1	0.9	4	51.54	1	706	4	104	8	69	2.4	2.00		0.44	0.47	0.01	0.89	0.01	0.84	1.12	0.85	0.029690924
14	73	0	25.95	1	0.84	2	31.89	1	1444	4	166	10	74	2.72	8.00		0.72	0.77	0.02	1.45	0.02	1.37	1.82	1.39	0.048378973
15	71	0	30	1	0.84	1	218.02	1	559	3	54	0.67	77	2.73	2.00		0.05	0.06	0	0.11	0	0.1	0.13	0.1	0.003504912
16	58	0	26.28	1	1.14	2	200.45	1	731	4	131	2.35	63	2.4	1.00		0.11	0.12	0	0.22	0	0.21	0.27	0.21	0.007270839
17	67	1	23.71	0	1.03	1	25.58	0	766	1	37	2	64	2.4	3.00		0.1	0.1	0	0.19	0	0.18	0.24	0.18	0.00638595
18	54	1	29.41	1	0.79	3	393.08	1	1291	1	92	0.48	67	2.4	1.00		0.03	0.03	0	0.05	0	0.05	0.06	0.05	0.001691344
19	64	2	29.22	0	0.8	1	53.61	1	836	4	26	0.47	64	2.4	1.00		0.02	0.02	0	0.04	0	0.04	0.06	0.04	0.001490055
20	78	0	36.12	1	1.24	1	222.64	1	611	4	100	0.47	64	2.4	2.00		0.02	0.02	0	0.04	0	0.04	0.06	0.04	0.001490055
21	54	2	15.21	0	0.57	4	26.68	1	1435	4	78	4.48	62	1.9	4.00		0.16	0.17	0.01	0.32	0.01	0.3	0.4	0.31	0.010635608
22	57	2	22.55	0	0.6	1	90.04	1	952	4	90	6	69	2.4	1.00		0.33	0.35	0.01	0.67	0.01	0.63	0.84	0.64	0.022268193
23	64	0	23.96	3	0.71	2	131.74	1	1102	1	96	7	68	2.22	2.00		0.35	0.37	0.01	0.7	0.01	0.66	0.88	0.67	0.023339585
24	36	1	22.72	0	0.96	4	148.41	1	1040	4	125	8	71	2.4	2.00		0.47	0.5	0.02	0.94	0.02	0.89	1.18	0.9	0.031437082
25	82	1	25.61	1	1.45	4	5.17	0	180	4	66	4	77	2.82	2.00		0.32	0.35	0.01	0.65	0.01	0.61	0.82	0.63	0.021722752
26	70	2	28.26	0	0.79	1	26.68	0	685	1	39	2	68	2.4	1.00		0.11	0.11	0	0.22	0	0.2	0.27	0.21	0.007209138
27	32	1	25.46	2	1.02	3	626.79	1	548	4	86	6	69	2.94	2.00		0.41	0.43	0.01	0.82	0.01	0.77	1.03	0.79	0.027278536
28	82	0	28.61	1	0.83	2	24.62	0	446	2	31	0.35	77.5	2.79	1.00		0.03	0.03	0	0.06	0	0.05	0.07	0.05	0.001905022
29	61	1	27.8	3	0.8	1	21.26	1	653	1	32	1	65	2.8	11.00		0.06	0.06	0	0.12	0	0.11	0.14	0.11	0.003842457
30	58	1	28.82	1	0.93	3	464.04	0	1454	4	92	7	68	2.94	3.00		0.46	0.49	0.02	0.93	0.02	0.87	1.16	0.89	0.030909181
31	45	1	24.22	1	0.86	2	162.78	1	1564	1	71		67	2.4	1.00		0	0	0	0	0	0	0	0	0
32	65	0	23.72	3	0.71	3	21.8	0	491	3	24.51	0.32	63	2.4	1.00		0.01	0.02	0	0.03	0	0.03	0.04	0.03	0.000979758
33	47	0	18.74	1	0.71	4	41.85	1	935	4	92	7	68	2.4	2.00		0.38	0.4	0.01	0.76	0.01	0.71	0.95	0.73	0.025231984
34	49	0	22.89	1	0.47	2	19.26	1	570	1	190	10	75	2.72	2.00		0.74	0.79	0.02	1.49	0.02	1.41	1.87	1.43	0.049695347
35	72	0	24.8	3	0.98	2	200.73	0	1158	1	60	4	66	2.82	9.00		0.24	0.25	0.01	0.48	0.01	0.45	0.6	0.46	0.015959573
36	43	1	26.18	0	0.83	1	57.63	1	1037	2	84	1.67	80	2.94	1.00		0.15	0.16	0.01	0.31	0.01	0.29	0.38	0.29	0.010185922
37	69	0	32.6	3	0.82	2	196.36	0	374	1	38	0.32	68	2.4	2.00		0.02	0.02	0	0.03	0	0.03	0.04	0.03	0.001141447
38	46	1	25.62	0	1.29	2	76.54	0	1244	1	126	7	71	2.4	1.00		0.41	0.44	0.01	0.82	0.01	0.78	1.04	0.79	0.027507447
39	72	0	24.8	3	0.99	4	213.67	1	1095	3	120	8	71	2.4	4.00		0.47	0.5	0.02	0.94	0.02	0.89	1.18	0.9	0.031437082
40	40	1	30.42	1	0.75	3	173.64	1	555	1	72	1.35	65	2.4	2.00		0.07	0.07	0	0.13	0	0.13	0.17	0.13	0.004446272
41	38	2	19.71	0	0.69	4	35.22	1	817	2	45	1.17	67.5	2.18	2.00		0.06	0.06	0	0.11	0	0.11	0.14	0.11	0.003763874
42	50	2	20.54	0	0.33	2	22.03	1	309	2	185	10	75	2.72	2.00		0.74	0.79	0.02	1.49	0.02	1.41	1.87	1.43	0.049695347
43	69	1	21.48	0	0.76	2	245.14	1	1311	4	85	16.68	64	2.4	16.00		0.79	0.85	0.03	1.6	0.03	1.51	2.01	1.53	0.053269463
44	47	0	18.74	1	0.67	4	34.44	0	837	4	102	8	69	2.4	2.00		0.44	0.47	0.01	0.89	0.01	0.84	1.12	0.85	0.029690924
45	59	1	25.16	0	0.71	3	345.57	1	1515	3	98	8	68	2.22	1.00		0.4	0.42	0.01	0.8	0.01	0.75	1	0.77	0.026673812
46	61	1	25.28	1	0.83	4	45.09	0	1373	3	102	8	69	2.4	1.00		0.44	0.47	0.01	0.89	0.01	0.84	1.12	0.85	0.029690924
47	61	1	19.2	0	0.66	1	13.66	1	341	2	27	1	64	2.4	2.00		0.05	0.05	0	0.1	0	0.09	0.12	0.09	0.003192975
48	55	0	20.03	1	0.76	1	64.25	0	1197	1	72	4	67	2.64	2.00		0.23	0.24	0.01	0.46	0.01	0.44	0.58	0.44	0.015397061
49	59	1	29.11	0	1.16	3	47.19	1	595	4	104	8	69	2.4	1.00		0.44	0.47	0.01	0.89	0.01	0.84	1.12	0.85	0.029690924
50	47	1	24.98	0	1.05	4	33.52	1	766	4	63	2.12	77	2.82	1.00		0.17	0.18	0.01	0.34	0.01	0.33	0.43	0.33	0.011494956
51	48	2	19.72	0	0.65	1	124.6	1	1688	1	98	7	68	2.22	1.00		0.35	0.37	0.01	0.7	0.01	0.66	0.88	0.67	0.023339585
52	72	1	24.8	3	0.96	4	96.29	1	1023	4	65	4	77	2.82	2.00		0.32	0.35	0.01	0.65	0.01	0.61	0.82	0.63	0.021722752
53	58	0	26.28	1	1.19	3	27.23	1	614	4	59	0.67	64	2.4	1.00		0.03	0.03	0	0.06	0	0.06	0.08	0.06	0.00212865
54	48	2	21.53	0	0.65	4	49.6	1	645	2	45	0.67	64	2.4	2.00		0.03	0.03	0	0.06	0	0.06	0.08	0.06	0.00212865
55	31	1	22.86	0	1.24	4	30.24	1	179	4	112	8	69	2.4	1.00		0.44	0.47	0.01	0.89	0.01	0.84	1.12	0.85	0.029690924
56	44	2	22.48	0	0.7	4	85.44	1	822	3	120	8	70	2.4	6.00		0.46	0.49	0.02	0.92	0.02	0.86	1.15	0.88	0.030557766
57	40	1	37.66	0	0.95	2	256.51	0	1451	1	51	3	68	2.4	2.00		0.16	0.17	0.01	0.32	0.01	0.31	0.41	0.31	0.010813708
58	46	1	25.62	0	1.13	4	35.87	1	1320	2	78	4.2	67	2.4	1.00		0.22	0.23	0.01	0.44	0.01	0.42	0.55	0.42	0.014697194
59	73	2	22.05	0	0.7	2	56.42	0	1415	4	136	9	73	2.72	2.00		0.63	0.67	0.02	1.27	0.02	1.2	1.6	1.22	0.042372241
60	59	1	25.16	0	1.05	2	177.49	1	1487	4	69	6	67	2.4	1.00		0.31	0.33	0.01	0.63	0.01	0.59	0.79	0.6	0.020995992
61	78	1	30.48	1	1.04	1	142.26	0	1721	1	77	3.12	79	2.87	1.00		0.27	0.29	0.01	0.54	0.01	0.51	0.68	0.52	0.01813221
62	24	1	20.42	0	0.78	1	67.27	0	1007	1	25.7	0.52	73	2.64	1.00		0.04	0.04	0	0.07	0	0.07	0.09	0.07	0.002360937
63	71	1	25.45	2	1.95	4	89.48	1	533	2	66	1.93	67	2.4	2.00		0.1	0.11	0	0.2	0	0.19	0.25	0.19	0.006765375
64	35	1	26.53	0	1.13	1	52.94	0	1104	1	59	4	65	2.82	3.00		0.23	0.25	0.01	0.46	0.01	0.44	0.58	0.45	0.015479613
65	62	2	20.6	0	0.82	2	14.89	1	1030	4	35	1.2	73	2.61	1.00		0.08	0.09	0	0.16	0	0.15	0.2	0.16	0.005421154
66	72	0	34.63	1	0.64	3	115.4	1	436	4	106	8	69	2.4	4.00		0.44	0.47	0.01	0.89	0.01	0.84	1.12	0.85	0.029690924
67	60	1	31.79	0	1.19	4	442	1	1005	3	120	8	71	2.4	7.00		0.47	0.5	0.02	0.94	0.02	0.89	1.18	0.9	0.031437082
68	63	2	20.32	0	0.6	2	402.49	1	1167	4	32	0.7	67	2.4	6.00		0.04	0.04	0	0.07	0	0.07	0.09	0.07	0.002449532
69	72	2	23.9	0	1.01	2	24.02	1	1020	2	180	10	74	2.72	1.00		0.72	0.77	0.02	1.45	0.02	1.37	1.82	1.39	0.048378973
70	47	1	32.11	0	1.11	3	476.62	1	538	3	108	8	69	2.4	1.00		0.44	0.47	0.01	0.89	0.01	0.84	1.12	0.85	0.029690924
71	20	1	19	0	0.97	1	95.42	1	625	1	94	6	68	2.4	2.00		0.32	0.34	0.01	0.65	0.01	0.61	0.81	0.62	0.021627415
72	69	2	21.19	2	0.62	2	10.02	0	606	3	180	10	74	2.72	2.00		0.72	0.77	0.02	1.45	0.02	1.37	1.82	1.39	0.048378973
73	79	0	20.31	1	0.56	1	78.07	0	1013	4	84	5	69	2.15	5.00		0.25	0.26	0.01	0.5	0.01	0.47	0.63	0.48	0.016623824
74	44	2	22.48	0	0.92	1	74.5	0	504	2					2.00		0	0	0	0	0	0	0	0	0
75	65	1	26.22	1	0.81	4	47.16	0	722	4	92	0.48	67	2.4	1.00		0.03	0.03	0	0.05	0	0.05	0.06	0.05	0.001691344
76	41	2	25.15	0	0.51	4	23.94	1	553	4	76	6	68	2.64	3.00		0.35	0.38	0.01	0.71	0.01	0.67	0.9	0.68	0.023790157
77	45	1	25.17	0	1.09	1	5.26	1	897	4	26	1	64	2.64	1.00		0.05	0.06	0	0.11	0	0.1	0.13	0.1	0.003512272
78	48	2	30.19	0	0.83	2	129.94	0	1097	1	160	2.2	67	2.4	2.00		0.11	0.12	0	0.23	0	0.22	0.29	0.22	0.00769853
79	74	1	22.07	0	0.79	1	221.29	1	1459	1	98	8	69	2.22	2.00		0.41	0.44	0.01	0.82	0.01	0.78	1.03	0.79	0.027464104
80	61	0	27.04	1	0.78	1	58.07	0	1194	4	68	4	67	2.4	3.00		0.21	0.22	0.01	0.42	0.01	0.4	0.53	0.4	0.013997328
81	42	2	26.29	2	0.76	2	132.45	1	1087	1	78	1.65	76	2.72	1.00		0.13	0.13	0	0.25	0	0.24	0.32	0.24	0.00841985
82	36	1	24.96	2	0.78	2	133.4	1	1323	3	136	3.05	65	2.4	1.00		0.15	0.16	0.01	0.3	0.01	0.28	0.38	0.29	0.010045281
83	48	2	30.19	0	0.77	1	114.34	1	868	4	97	7	68	2.22	1.00		0.35	0.37	0.01	0.7	0.01	0.66	0.88	0.67	0.023339585
84	69	0	28.64	1	0.91	3	45.5	0	313	4					2.00		0	0	0	0	0	0	0	0	0
85	51	1	43.75	1	0.82	1	91.81	1	1381	2	92	6	68	2.4	2.00		0.32	0.34	0.01	0.65	0.01	0.61	0.81	0.62	0.021627415
86	72	0	27.77	1	0.52	1	38.92	1	1122	1	32	0.22	67	2.4	2.00		0.01	0.01	0	0.02	0	0.02	0.03	0.02	0.000758189
87	46	1	24.53	0	0.94	3	33.69	1	1422	4	112	8	70	2.4	1.00		0.46	0.49	0.02	0.92	0.02	0.86	1.15	0.88	0.030557766
88	37	1	27.17	0	0.78	2	79.39	1	1021	1	116	8	70	2.4	1.00		0.46	0.49	0.02	0.92	0.02	0.86	1.15	0.88	0.030557766
89	38	0	26.56	1	0.47	1	39.35	1	1118	1	47	3	68	2.18	2.00		0.15	0.16	0	0.29	0	0.28	0.37	0.28	0.009822451
90	59	2	26.91	0	0.79	1	35.39	0	1270	1	43	2	67	2.4	2.00		0.1	0.11	0	0.21	0	0.2	0.26	0.2	0.006998664
91	45	1	29.08	3	1.06	2	30.54	1	1045	1	171	10	74	2.72	1.00		0.72	0.77	0.02	1.45	0.02	1.37	1.82	1.39	0.048378973
92	73	1	22.17	1	1.15	4	82.09	0	1219	2	118	8	70	2.4	2.00		0.46	0.49	0.02	0.92	0.02	0.86	1.15	0.88	0.030557766
93	65	2	27.24	2	0.83	1	36.97	0	994	1	45	3	67	2.18	1.00		0.14	0.15	0	0.29	0	0.27	0.36	0.27	0.00953568
94	58	1	25.88	3	0.92	2	79.03	1	718	4	121	7	71	2.4	1.00		0.41	0.44	0.01	0.82	0.01	0.78	1.04	0.79	0.027507447
95	63	2	24.77	0	0.66	2	24.28	0	781	2	177	10	74	2.72	2.00		0.72	0.77	0.02	1.45	0.02	1.37	1.82	1.39	0.048378973
96	51	2	21.94	0	0.57	1	196.51	1	918	3	84	4	67	2.15	2.00		0.19	0.2	0.01	0.38	0.01	0.35	0.47	0.36	0.012539273
97	51	2	20.17	0	0.44	4	34.29	1	1067	4	96	0.67	67.5	2.22	1.00		0.03	0.03	0	0.07	0	0.06	0.08	0.06	0.002190249
98	43	2	30.86	0	0.49	1	42.14	1	1024	1	48	3	68	2.18	1.00		0.15	0.16	0	0.29	0	0.28	0.37	0.28	0.009822451
99	51	1	19.82	1	0.89	2	33.31	0	1314	1	165	10	74	2.72	1.00		0.72	0.77	0.02	1.45	0.02	1.37	1.82	1.39	0.048378973
100	25	2	18.73	0	0.6	3	6.8	0	290	3	118	8	70	2.4	3.00		0.46	0.49	0.02	0.92	0.02	0.86	1.15	0.88	0.030557766
101	33	1	28.52	0	0.91	2	39.88	1	1029	2	145	9	73	2.72	2.00		0.63	0.67	0.02	1.27	0.02	1.2	1.6	1.22	0.042372241
102	56	2	26.28	2	0.48	3	54.08	1	1325	4	124	6	71	2.4	2.00		0.35	0.37	0.01	0.71	0.01	0.67	0.89	0.68	0.023577811
103	64	1	21.39	3	0.41	1	79.54	0	1464	1	86	5	69	2.94	1.00		0.34	0.36	0.01	0.68	0.01	0.64	0.86	0.65	0.022732113
104	77	2	29.47	2	0.83	4	22.16	0	435	4	120	8	70	2.4	2.00		0.46	0.49	0.02	0.92	0.02	0.86	1.15	0.88	0.030557766
105	71	1	22.72	1	1.61	2	43.28	1	188	4	140	9	73	2.72	2.00		0.63	0.67	0.02	1.27	0.02	1.2	1.6	1.22	0.042372241
106	58	1	25.83	2	0.83	2	175.84	1	1461	1	70	6	67	2.4	1.00		0.31	0.33	0.01	0.63	0.01	0.59	0.79	0.6	0.020995992
107	64	2	23.68	0	1.07	3	93.19	1	1371	4	130	8	71	2.4	2.00		0.47	0.5	0.02	0.94	0.02	0.89	1.18	0.9	0.031437082
108	57	1	26.89	0	0.67	3	94.82	1	1309	4	136	8	71	2.72	1.00		0.53	0.57	0.02	1.07	0.02	1.01	1.34	1.03	0.035628693
109	33	2	26.17	0	0.48	4	58.63	1	936	4	106	8	69	2.4	1.00		0.44	0.47	0.01	0.89	0.01	0.84	1.12	0.85	0.029690924
110	55	2	24.26	0	0.51	1	71.67	0	1041	1	79	5	76	2.72	3.00		0.38	0.41	0.01	0.76	0.01	0.72	0.96	0.73	0.025514696
111	79	1	21.04	1	0.83	1	183.28	0	840	1	95	7	68	2.4	4.00		0.38	0.4	0.01	0.76	0.01	0.71	0.95	0.73	0.025231984
112	48	1	26.88	0	0.86	3	113.28	1	755	4	142	9	73	2.72	1.00		0.63	0.67	0.02	1.27	0.02	1.2	1.6	1.22	0.042372241
113	56	2	27.15	0	0.62	1	149.96	1	1930	1	127	2.32	67	2.4	2.00		0.12	0.13	0	0.24	0	0.23	0.31	0.23	0.008106786
114	58	1	25.83	2	0.83	1	165.93	1	1499	1	94	7	68	2.4	1.00		0.38	0.4	0.01	0.76	0.01	0.71	0.95	0.73	0.025231984
115	56	1	23.54	0	0.97	1	94.21	1	1500	4	93	6	68	2.4	3.00		0.32	0.34	0.01	0.65	0.01	0.61	0.81	0.62	0.021627415
116	55	1	25.45	3	1.14	2	194.1	1	1546	1	65	4	77	2.82	2.00		0.32	0.35	0.01	0.65	0.01	0.61	0.82	0.63	0.021722752
117	60	2	27.37	0	0.47	3	86.38	1	677	1	148	9	73	2.72	1.00		0.63	0.67	0.02	1.27	0.02	1.2	1.6	1.22	0.042372241
118	38	1	31.2	0	1	1	54.58	1	789	1	60	4	66	2.82	2.00		0.24	0.25	0.01	0.48	0.01	0.45	0.6	0.46	0.015959573
119	67	1	31.24	1	0.79	4	109.93	1	1251	4	120	8	70	2.4	2.00		0.46	0.49	0.02	0.92	0.02	0.86	1.15	0.88	0.030557766
120	54	2	21.13	0	0.51	1	208.05	0	1358	1	96	8	68	2.22	7.00		0.4	0.42	0.01	0.8	0.01	0.75	1	0.77	0.026673812
121	42	2	35.43	0	0.77	1	17.64	1	529	1	28	1	64	2.3	2.00		0.05	0.05	0	0.09	0	0.09	0.12	0.09	0.003059934
122	37	2	45.54	0	0.67	1	120.97	1	933	1	98	7	68	2.22	2.00		0.35	0.37	0.01	0.7	0.01	0.66	0.88	0.67	0.023339585
123	59	1	22.27	3	0.72	2	90.96	0	1525	4	106	8	69	2.4	2.00		0.44	0.47	0.01	0.89	0.01	0.84	1.12	0.85	0.029690924
124	67	1	31.24	1	0.64	3	15.83	1	460	4	152	9	73	2.72	1.00		0.63	0.67	0.02	1.27	0.02	1.2	1.6	1.22	0.042372241
125	56	0	29.14	1	0.63	1	36.97	1	619	1	44	2	67	2.4	2.00		0.1	0.11	0	0.21	0	0.2	0.26	0.2	0.006998664
126	61	1	22.76	0	0.96	1	37.69	1	1825	4	46	3	68	2.18	1.00		0.15	0.16	0	0.29	0	0.28	0.37	0.28	0.009822451
127	67	1	23.62	0	0.95	4	41.92	0	870	4	98	8	68	2.22	2.00		0.4	0.42	0.01	0.8	0.01	0.75	1	0.77	0.026673812
128	54	1	24.72	1	0.9	1	137.66	1	1339	1	93	7	68	2.4	2.00		0.38	0.4	0.01	0.76	0.01	0.71	0.95	0.73	0.025231984
129	69	0	28.64	1	0.95	3	45.94	1	1202	3					4.00		0	0	0	0	0	0	0	0	0
130	51	2	24.11	0	0.6	3	33.48	0	238	3	150	9	73	2.72	2.00		0.63	0.67	0.02	1.27	0.02	1.2	1.6	1.22	0.042372241
131	66	2	27.19	0	0.48	2	57.76	1	867	1	131	7	71	2.4	1.00		0.41	0.44	0.01	0.82	0.01	0.78	1.04	0.79	0.027507447
132	47	2	23.63	0	0.57	1	46.93	0	930	4	42	1.05	67	2.4	2.00		0.05	0.06	0	0.11	0	0.1	0.14	0.11	0.003674299
133	48	0	27.25	3	0.68	4	202.77	0	1495	1	125	6	71	2.4	3.00		0.35	0.37	0.01	0.71	0.01	0.67	0.89	0.68	0.023577811
134	63	0	33.89	1	0.5	1	18.94	1	1016	1	29	1	64	2.4	1.00		0.05	0.05	0	0.1	0	0.09	0.12	0.09	0.003192975
135	57	2	29.69	0	0.73	1	72.83	1	1331	4	80	5	76	2.72	2.00		0.38	0.41	0.01	0.76	0.01	0.72	0.96	0.73	0.025514696
136	58	1	26.3	1	0.82	2	482.63	0	1243	4	36		73	2.6	4.00		0	0	0	0	0	0	0	0	0
137	35	1	21.22	1	0.39	2	82.47	0	948	4	111	8	69	2.4	5.00		0.44	0.47	0.01	0.89	0.01	0.84	1.12	0.85	0.029690924
138	60	0	24.15	1	0.69	1	65.65	1	1008	1	73	4	68	2.64	1.00		0.24	0.25	0.01	0.48	0.01	0.45	0.6	0.46	0.015860104
139	60	1	25.63	0	2.6	4	196.86	1	426	1	120	8	70	2.4	2.00		0.46	0.49	0.02	0.92	0.02	0.86	1.15	0.88	0.030557766
140	54	0	25.46	3	0.71	2	156.77	0	1819	1	86	6	69	2.94	7.00		0.41	0.43	0.01	0.82	0.01	0.77	1.03	0.79	0.027278536
141	60	2	26.53	0	0.6	2	34.12	1	555	1	160	10	73	2.72	1.00		0.7	0.75	0.02	1.41	0.02	1.33	1.77	1.36	0.047080268
142	62	2	19.95	0	0.66	3	13.31	1	538	1	140	9	73	2.72	2.00		0.63	0.67	0.02	1.27	0.02	1.2	1.6	1.22	0.042372241
143	31	1	23.5	0	0.98	1	122.31	1	666	2	99	7	69	2.22	2.00		0.36	0.38	0.01	0.72	0.01	0.68	0.9	0.69	0.024031091
144	57	1	26.87	1	1.96	1	85.73	0	1415	4	88	6	69	2.4	2.00		0.33	0.35	0.01	0.67	0.01	0.63	0.84	0.64	0.022268193
145	77	2	23.61	0	0.89	1	23.94	1	1060	4	35	3	67	2.4	1.00		0.16	0.17	0.01	0.31	0.01	0.3	0.4	0.3	0.010497996
146	45	2	20.06	0	0.49	1	69.9	1	897	4	76	4	68	2.64	1.00		0.24	0.25	0.01	0.48	0.01	0.45	0.6	0.46	0.015860104
147	42	1	23.63	0	0.89	1	25.56	0	654	3	36	2	73	2.6	2.00		0.13	0.14	0	0.27	0	0.25	0.34	0.26	0.009000639
148	41	1	26.26	0	1.02	1	43.61	0	1220	1	50	3	68	2.4	2.00		0.16	0.17	0.01	0.32	0.01	0.31	0.41	0.31	0.010813708
149	78	0	22.51	1	1.14	1	75.3	0	925	1	82	5	76	2.72	7.00		0.38	0.41	0.01	0.76	0.01	0.72	0.96	0.73	0.025514696
150	47	1	22.92	0	0.93	1	32.61	1	398	1	42	2	68	2.4	2.00		0.11	0.11	0	0.22	0	0.2	0.27	0.21	0.007209138
151	61	1	30.88	0	0.93	2	128.17	1	744	4	101	8	69	2.4	2.00		0.44	0.47	0.01	0.89	0.01	0.84	1.12	0.85	0.029690924
152	55	0	24.26	1	1.04	1	61.69	1	1518	4	69	4	67	2.4	1.00		0.21	0.22	0.01	0.42	0.01	0.4	0.53	0.4	0.013997328
153	84	2	24.61	0	0.49	1	101.17	0	1022	1	96	6	68	2.22	1.00		0.3	0.32	0.01	0.6	0.01	0.57	0.75	0.58	0.020005359
154	36	2	21.91	0	0.79	1	43.25	1	515	4	49	3	68	2.4	1.00		0.16	0.17	0.01	0.32	0.01	0.31	0.41	0.31	0.010813708
155	53	1	2.48	3	0.65	1	71.03	0	914	1	77	4	72	2.4	1.00		0.24	0.26	0.01	0.48	0.01	0.46	0.61	0.47	0.016164435
156	72	1		0	0.83	1	314.2	1	532	2	102	8	69	2.4	7.00		0.44	0.47	0.01	0.89	0.01	0.84	1.12	0.85	0.029690924
157	50	2	25.53	0	0.85	2	162.07	1	1780	4	76	6	68	2.64	2.00		0.35	0.38	0.01	0.71	0.01	0.67	0.9	0.68	0.023790157
158	72	1		0	1.48	1	267.85	0	518	1	101	8	69	2.4	2.00		0.44	0.47	0.01	0.89	0.01	0.84	1.12	0.85	0.029690924
159	71	0	28.19	3	0.83	1	65.73	0	528	1	75	4	68	2.64	1.00		0.24	0.25	0.01	0.48	0.01	0.45	0.6	0.46	0.015860104
160	53	1	25.16	3	0.69	2	291.91	0	877	4	41	2	68	2.4	1.00		0.11	0.11	0	0.22	0	0.2	0.27	0.21	0.007209138
161	58	1		0	0.88	2	240.51	0	473	4	55		77	2.7	1.00		0	0	0	0	0	0	0	0	0
162	79	0	22.27	1	1.03	1	96.72	0	1312	1	95	6	68	2.4	1.00		0.32	0.34	0.01	0.65	0.01	0.61	0.81	0.62	0.021627415
163	57	1	26.17	0	0.67	2	69.08	0	632	4	130	6	71	2.4	1.00		0.35	0.37	0.01	0.71	0.01	0.67	0.89	0.68	0.023577811
164	63	1	20.2	0	0.82	2	100.91	1	1461	1	76	2	72	2.64	1.00		0.13	0.14	0	0.27	0	0.25	0.33	0.26	0.008890439
165	72	1	27.89	1	1.47	1	52.59	0	839	1	56.19	2.16	68	2.4	2.00		0.12	0.12	0	0.23	0	0.22	0.29	0.22	0.007785869
166	54	1	28.34	3	0.73	1	420.26	0	950	4	71	1.31	66	2.4	2.00		0.07	0.07	0	0.13	0	0.13	0.17	0.13	0.004448307
167	66	0	24.89	1	0.5	1	22.71	1	616	1	34	1	67	2.4	1.00		0.05	0.06	0	0.1	0	0.1	0.13	0.1	0.003499332
168	40	1	22.78	0	0.71	1	86.72	0	1480	1	56.19	0.51	65	2.4	1.00		0.03	0.03	0	0.05	0	0.05	0.06	0.05	0.001679703
169	49	1	22.72	2	1.51	2	457.48	1	424	4	107.14	2.22	64	2.4	1.00		0.11	0.11	0	0.21	0	0.2	0.27	0.2	0.007088404
170	58	1	26.67	0	0.97	1	25.19	0	605	1	30.2	2.07	64	2.4	2.00		0.1	0.11	0	0.2	0	0.19	0.25	0.19	0.006609458
171	54	1	25.62	0	0.91	1	28.21	0	699	1	48.31	4.22	66	2.4	1.00		0.21	0.23	0.01	0.43	0.01	0.41	0.54	0.41	0.014329659
172	35	1	27.73	0	1.8	1	127.36	1	1617	4	91	7	68	2.4	4.00		0.38	0.4	0.01	0.76	0.01	0.71	0.95	0.73	0.025231984
173	28	1	21.11	0	0.88	1	78.76	0	1364	1	85	5	69	2.94	2.00		0.34	0.36	0.01	0.68	0.01	0.64	0.86	0.65	0.022732113
174	56	1	29.05	0	1.22	1	21.56	0	623	1	33	1	67	2.4	2.00		0.05	0.06	0	0.1	0	0.1	0.13	0.1	0.003499332
175	74	1	26.33	0	0.75	1	90.91	0	1463	1	91	6	68	2.4	2.00		0.32	0.34	0.01	0.65	0.01	0.61	0.81	0.62	0.021627415
176	66	2	22.67	0	0.78	1	89.68	0	644	4	56.5	1.47	61	2.4	1.00		0.06	0.07	0	0.13	0	0.12	0.16	0.12	0.004263954
177	37	1	25.59	0	0.84	1	50.79	0	1150	3	52	1.57	73	2.52	5.00		0.1	0.11	0	0.21	0	0.19	0.26	0.2	0.006848102
178	63	1	28.65	0	1	1	46.69	0	532	1	55	3	77	2.7	2.00		0.23	0.25	0.01	0.47	0.01	0.44	0.59	0.45	0.015598785
179	58	1	27.4	0	0.81	1	3.86	0	425	3	25	1	64	2.4	3.00		0.05	0.05	0	0.1	0	0.09	0.12	0.09	0.003192975
180	47	1	29.07	0	0.8	1	133.8	1	1542	4	92	7	68	2.4	1.00		0.38	0.4	0.01	0.76	0.01	0.71	0.95	0.73	0.025231984
181	67	1	22.05	0	3.45	1	217.41	1	326	4	97	8	68	2.22	2.00		0.4	0.42	0.01	0.8	0.01	0.75	1	0.77	0.026673812
182	57	1	29.41	0	1.13	2	369.69	0	786	2	122	2.46	67	2.4	2.00		0.13	0.14	0	0.26	0	0.24	0.32	0.25	0.008608357
183	59	1	28.41	0	1.09	1	147.53	1	1290	1	90	1.26	67	2.4	2.00		0.07	0.07	0	0.13	0	0.12	0.17	0.13	0.004409158
184	59	1	23.03	2	0.99	1	71.06	1	1134	4	36	6.06	64	2.4	1.00		0.29	0.31	0.01	0.58	0.01	0.55	0.73	0.56	0.019349427
185	54	2	24.22	0	0.86	1	40.94	0	1114	1	50	0.26	63	2.4	3.00		0.01	0.01	0	0.02	0	0.02	0.03	0.02	0.000804433
186	62	1	24.22	0	1.79	2	319.98	0	582	1	60	0.26	63	2.4	3.00		0.01	0.01	0	0.02	0	0.02	0.03	0.02	0.000804433
187	44	1		0	1.03	1	45.59	0	688	4	23.33	0.45	65	2.4	2.00		0.02	0.02	0	0.04	0	0.04	0.06	0.04	0.001482091
188	55	1	24.39	0	0.95	1	52.49	1	852	3	77	1.07	65	2.4	1.00		0.05	0.06	0	0.11	0	0.1	0.13	0.1	0.003524082
189	67	2	24.34	0	0.87	1	81.3	0	1060	1	27.13	10	61	2.3	1.00		0.41	0.44	0.01	0.83	0.01	0.79	1.05	0.8	0.027797888
190	57	1	19.95	0	1.36	1	51.15	0	1159	1	58	3	77	2.7	1.00		0.23	0.25	0.01	0.47	0.01	0.44	0.59	0.45	0.015598785
191	40	2	31.18	0	0.61	1	19.27	1	778	1	30	1	64	2.4	3.00		0.05	0.05	0	0.1	0	0.09	0.12	0.09	0.003192975
192	42	2	28.28	0	0.56	1	331.57	1	1072	3	103	8	69	2.4	3.00		0.44	0.47	0.01	0.89	0.01	0.84	1.12	0.85	0.029690924
193	45	1	28.86	0	0.9	3	698.96	1	864	3	60	4	66	2.82	2.00		0.24	0.25	0.01	0.48	0.01	0.45	0.6	0.46	0.015959573
194	43	1	28.71	0	0.98	2	154.45	0	1136	3	91	7	70	2.4	1.00		0.4	0.43	0.01	0.8	0.01	0.76	1.01	0.77	0.026738046
195	48	1	22.95	0	0.75	2	159.38	1	909	3	81	6	76	2.72	1.00		0.46	0.49	0.02	0.92	0.02	0.87	1.15	0.88	0.030617635
196	45	2	21.05	0	0.71	1	19.75	0	174	1	31	1	65	2.7	2.00		0.06	0.06	0	0.11	0	0.1	0.14	0.11	0.003705227
197	64	2	19.11	0	1.18	1	23.33	0	498	1	78	52	67	2.4	2.00		2.71	2.89	0.09	5.45	0.09	5.15	6.85	5.24	0.181965263
198	60	2	24.24	0	0.69	1	87.46	0	1654	1	89	6	69	2.4	2.00		0.33	0.35	0.01	0.67	0.01	0.63	0.84	0.64	0.022268193
199	55	1	24.39	0	1.12	1	54.59	1	1582	4	67	4	67	2.4	1.00		0.21	0.22	0.01	0.42	0.01	0.4	0.53	0.4	0.013997328
200	42	1	23.66	0	0.77	1	292.03	1	1372	4	120	1.09	62	2.4	1.00		0.05	0.05	0	0.1	0	0.09	0.12	0.09	0.00326622
201	57	2	26.17	0	0.78	2	797.68	0	1164	4	56	2.4	65	2.4	1.00		0.12	0.13	0	0.24	0	0.22	0.3	0.23	0.007904483
202	61	2	20.13	0	0.56	1	60.74	1	942	1	70.37	1.33	65	2.4	1.00		0.07	0.07	0	0.13	0	0.12	0.16	0.13	0.004380401
203	67	2	22.67	0	0.61	1	74.6	1	208	4	81	5	76	2.72	1.00		0.38	0.41	0.01	0.76	0.01	0.72	0.96	0.73	0.025514696
204	50	2	23.44	0	0.63	1	102.48	0	1469	3	30	1.33	65	2.4	2.00		0.07	0.07	0	0.13	0	0.12	0.16	0.13	0.004380401
205	53	1	26.53	1	1.07	1	35.08	0	1031	1	118.56	1.02	64	2.4	7.00		0.05	0.05	0	0.1	0	0.09	0.12	0.09	0.003256834
206	42	1	23.66	0	0.74	1	75.64	1	678	4	102.5	1.5	60	1.7	1.00		0.04	0.05	0	0.09	0	0.08	0.11	0.09	0.002981721
207	72	1	27.51	0	1.29	1	61.92	1	613	1	70	4	67	2.4	1.00		0.21	0.22	0.01	0.42	0.01	0.4	0.53	0.4	0.013997328
208	73	1	23.23	0	1.13	1	82.31	0	717	1	87	5	69	2.94	1.00		0.34	0.36	0.01	0.68	0.01	0.64	0.86	0.65	0.022732113
209	67	2	31.3	0	0.93	1	25.78	1	1889	1	38	2	64	2.4	1.00		0.1	0.1	0	0.19	0	0.18	0.24	0.18	0.00638595
210	39	1	33.69	0	0.79	1	47.18	1	1280	1	72	1.59	66	2.4	2.00		0.08	0.09	0	0.16	0	0.15	0.2	0.16	0.00539909
211	44	1	28.58	0	0.95	1	31.71	1	886	1	41	2	68	2.4	2.00		0.11	0.11	0	0.22	0	0.2	0.27	0.21	0.007209138
212	37	1	28.29	0	1.41	1	126.33	1	1197	1	90	7	69	2.4	1.00		0.39	0.41	0.01	0.78	0.01	0.73	0.98	0.75	0.025979558
213	49	1	25.25	0	0.97	2	277.08	0	832	4	46	3	68	2.18	1.00		0.15	0.16	0	0.29	0	0.28	0.37	0.28	0.009822451
214	81	1	20.96	0	1.42	2	822.47	1	552	4	31		65	2.4	2.00		0	0	0	0	0	0	0	0	0
215	63	2	25.15	0	0.48	1	168.85	0	1454	1	51.05	1.21	64	2.4	2.00		0.06	0.06	0	0.12	0	0.11	0.15	0.11	0.003863499
216	50	1	29.41	0	0.88	1	23.99	1	650	1	59	0.39	65	2.4	2.00		0.02	0.02	0	0.04	0	0.04	0.05	0.04	0.001284479
217	45	2	27.69	0	0.72	3	150.39	0	1228	4	53	2.23	65	2.4	1.00		0.11	0.12	0	0.22	0	0.21	0.28	0.21	0.007344583
218	65	1	26.18	1	0.62	1	72.67	1	1142	1	87.3	16.17	65	2.4	1.00		0.79	0.85	0.03	1.6	0.03	1.51	2.01	1.53	0.053256457
219	59	1	23.03	2	0.77	1	71.06	1	1134	4	78	5	79	2.72	1.00		0.41	0.44	0.01	0.83	0.01	0.78	1.04	0.79	0.02756877
220	54	1	25.1	0	0.89	1	232.19	1	1204	4	99	8	69	2.22	5.00		0.41	0.44	0.01	0.82	0.01	0.78	1.03	0.79	0.027464104
221	48	1	25.85	2	1.02	1	47.59	0	1025	1	56	3	77	2.7	1.00		0.23	0.25	0.01	0.47	0.01	0.44	0.59	0.45	0.015598785
222	71	2	22.48	0	0.7	1	30.13	0	487	1	40	2	68	2.4	2.00		0.11	0.11	0	0.22	0	0.2	0.27	0.21	0.007209138
223	63	1	25.51	2	0.88	1	179.75	0	1228	1	27	18	65	2.3	1.00		0.85	0.9	0.03	1.7	0.03	1.61	2.14	1.64	0.056813475
224	39	1	33.21	3	1.26	3	616.45	1	680	4	68	6	67	2.4	2.00		0.31	0.33	0.01	0.63	0.01	0.59	0.79	0.6	0.020995992
225	67	2	31.3	0	0.93	1	45.77	1	1456	1	51	3	68	2.4	2.00		0.16	0.17	0.01	0.32	0.01	0.31	0.41	0.31	0.010813708
226	58	1	25.01	1	0.95	1	1656.45	0	565	3	100	0.54	68	2.4	2.00		0.03	0.03	0	0.06	0	0.06	0.07	0.06	0.001946467
